# Study on the Spectrophotometric Detection of Free Fatty Acids in Palm Oil Utilizing Enzymatic Reactions

**DOI:** 10.3390/molecules200712328

**Published:** 2015-07-07

**Authors:** Nur Hidayah Azeman, Nor Azah Yusof, Jaafar Abdullah, Robiah Yunus, Mohd Nizar Hamidon, Reza Hajian

**Affiliations:** 1 Institute of Advanced Technology, Universiti Putra Malaysia, 43400 UPM Serdang, Selangor, Malaysia; E-Mails: dhaya.az687@gmail.com (N.H.A.); jafar@upm.edu.my (J.A.); robiah@upm.edu.my (R.Y.); mnh@upm.edu.my (M.N.H.); 2 Department of Chemistry, Faculty of Science, Universiti Putra Malaysia, 43400 UPM Serdang, Selangor, Malaysia; 3 Department of Chemical and Environmental Engineering, Faculty of Engineering, Universiti Putra Malaysia, 43400 UPM Serdang, Selangor, Malaysia; 4 Department of Electrical and Electronic Engineering, Faculty of Engineering, Universiti Putra Malaysia, 43400 UPM Serdang, Selangor, Malaysia

**Keywords:** free fatty acid, enzymatic reaction, aminolysis, palm oil, lipase, spectrophotometry determination

## Abstract

In this paper, a comprehensive study has been made on the detection of free fatty acids (FFAs) in palm oil via an optical technique based on enzymatic aminolysis reactions. FFAs in crude palm oil (CPO) were converted into fatty hydroxamic acids (FHAs) in a biphasic lipid/aqueous medium in the presence of immobilized lipase. The colored compound formed after complexation between FHA and vanadium (V) ion solution was proportional to the FFA content in the CPO samples and was analyzed using a spectrophotometric method. In order to develop a rapid detection system, the parameters involved in the aminolysis process were studied. The utilization of immobilized lipase as catalyst during the aminolysis process offers simplicity in the product isolation and the possibility of conducting the process under extreme reaction conditions. A good agreement was found between the developed method using immobilized *Thermomyces lanuginose* lipase as catalyst for the aminolysis process and the Malaysian Palm Oil Board (MPOB) standard titration method (R^2^ = 0.9453).

## 1. Introduction

Free fatty acid (FFA) detection in palm oil has attracted abundant attention from researchers since it is one of the most crucial aspects which influence the quality of palm oil products and their prices [1,2]. High FFA levels in palm oil lead to lower flavor quality [3], undesirable saponification, low product yields and complications in the subsequent separation processing steps [4]. Furthermore, a high FFA content in palm oil also may result in rancidity of the oil caused by the oxidation of unsaturated FFAs [5]. FFAs in palm oil can be increased in several ways which include damage to the palm fruits cells caused by the harvesting, rough handling or other processes [5], the action of enzymes in the palm fruits, the reaction of oil with water during storage [6] and lengthy storage of the palm fruits [7]. The Palm Oil Refiners Association of Malaysia (PORAM) has set standard specifications for the FFA content (as palmitic acid) which should be less than 5% and 0.1% in crude palm oil (CPO) and refined bleached deodorized oil (RBDO), respectively [6].

Traditionally, FFA levels in palm oil are determined using a manual titration method based on the Malaysian Palm Oil Board (MPOB) standard procedure which involves titrating the sample against potassium hydroxide (KOH) using phenolphthalein as indicator [8]. However, this method involves manual operations, requires a high amount of solvents and the presence of high amounts of carotene causes difficulties to determine the end point of the titration [2]. More advanced techniques used nowadays to determine FFAs in palm oil are capillary Gas Chromatography (GC), High Performance Liquid Chromatography (HPLC) [2], Fourier Transform Infrared Spectroscopy (FTIR) [6,9] and Near Infrared Spectroscopy (NIR) [10]. Although all of these techniques are based on high technology, the palm oil still has to be extracted prior to the analytical separation either through supercritical fluid extraction, Soxhlet extraction [11], liquid-liquid extraction or solid-phase extraction methods [2].

For determination of FFAs in various samples, the detection sensitivity can be improved by converting the FFAs into a derivative, such as a fatty acid methyl ester (FAME) [2,12]. In this study, the FFAs in palm oil were converted into fatty hydroxamic acids (FHAs) via an enzymatic route before optical detection in order to improve the assay sensitivity. This process is called an aminolysis process. The FHAs formed from in aminolysis process represent the original content of FFAs in palm oil since FFAs are intermediate compounds in the synthesis of FHAs [13]. Previous studies have shown that the synthesis of FHAs can be carried out via chemical synthesis [13] and enzymatic synthesis [14]. Enzymatic synthesis of FHAs has been conducted in biphasic lipid/aqueous media in the presence of lipase enzymes from various species such as *Candida parapsilopsis* [15], *Mucor miehei* [14], *Thermomyces lanuginose* [16] and *Rhizomucor meihei* [16,17].

In the aminolysis reaction of FFAs to FHAs, the enzyme is responsible for catalyzing the aminolysis of free fatty acids [15] in CPO. This type of enzyme is known as a triacylglycerol lipase [14]. Furthermore, since this reaction is carried out in a biphasic lipid/aqueous medium, lipase appears to be the most suitable enzyme because lipases naturally function at oil-water interfaces due to the fact their substrates are not soluble in water [18]. Moreover lipases are able to maintain their catalytic activity at low-water concentrations, such as those seen in organic phases, supercritical fluids and ionic media [19].

In this work, we used immobilized lipase in order to improve the stability of the enzyme when involved in extreme reaction conditions and for ease of product isolation [20]. On top of that, lipase catalysed reactions are a green and environmentally friendly technology [21,22]. The main objective of this study was to develop an alternative method for the detection of FFAs in crude palm oil based on an aminolysis method utilizing enzymes which uses less solvent and is environmentally friendly. The FHAs formed from the aminolysis of FFAs were reacted with V(V) ion solution in order to determine the original FFA concentration in the palm oil. The product complex was monitored using a spectrophotometric method. Basically, the principle of detection for FFA determination in CPO can be summarized as follows:

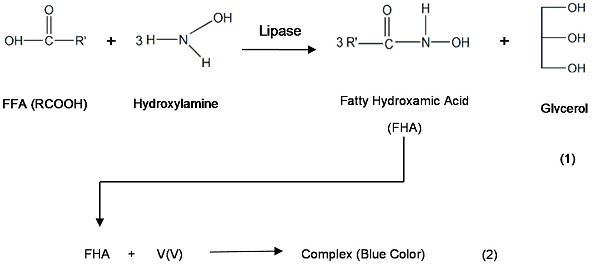


Equation (1) shows the chemical equation involved in the aminolysis of FFAs to FHAs. In order to ensure a fast detection system, it is necessary to study all the parameters involved in the aminolysis reaction before developing the detection system. Several parameters including time of reaction, mol ratio of the reactants, reaction temperature, types of enzymes and V(V) ion solution concentration were studied in order to ensure a significant and rapid colour change.

## 2. Results and Discussion

### 2.1. Detection of Free Fatty Acids (FFAs) Based on Enzymatic Aminolysis Reactions

The FFAs in palm oil can be converted into FHAs when reacted with hydroxylamine hydrochloride in the presence of lipase enzyme. This transformation was carried out in a biphasic lipid/aqueous medium. Hydroxylamine hydrochloride is a highly active nucleophile and acted as an acyl acceptor in this reaction [23]. FHAs are known as hydroxamic acid derivatives possessing chelating properties and able to form complexes with metal ions [24] such as V(V) [25], iron (III) [24] and copper (II) [26]. Thus the FHAs formed in the organic phase were reacted with V(V) to produce a blue coloured complex for spectrophotometric determination of the free fatty acids. This colored compound was proportional to the concentration of FFAs in palm oil and was analyzed via optical detection using a spectrophotometric technique. The important parameters for the quantitative reaction were type of enzymes, time of reaction and mole ratio of the reactants in order to obtain the most rapid and significant colour change for development of a FFA detection system.

**Figure 1:**
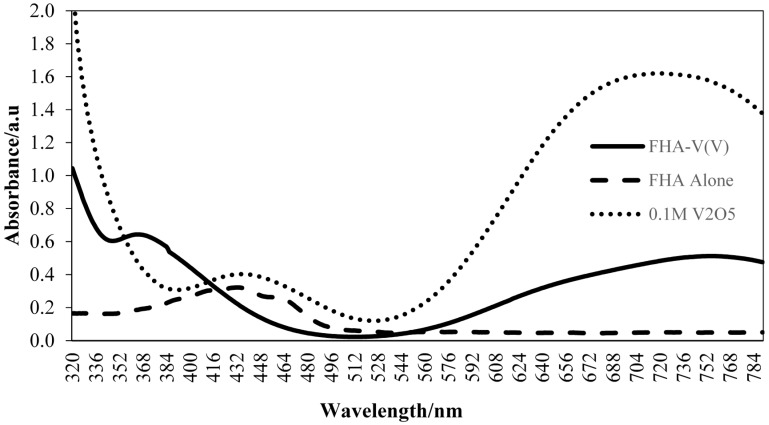
Absorbance spectra for aminolysis of FHA at 60 min of complexation with V(V) ion solution.

Figure 1 shows the absorbance spectra for FHA alone, FHA-V(V) complex and V(V) ion solution alone. The formation of the complex causes a shift in wavelength from right to the left due to a change in color of the reagent phase from yellow to blue. The maximum difference of the two absorbance spectra was observed at 364 nm and this wavelength was used for further analysis. The measurement was expressed as an absorbance difference, which is defined as the difference between the absorbance of the FHA alone and FHA-V(V) complex.

### 2.2. Effect of Aminolysis Reaction Time

**Figure 2:**
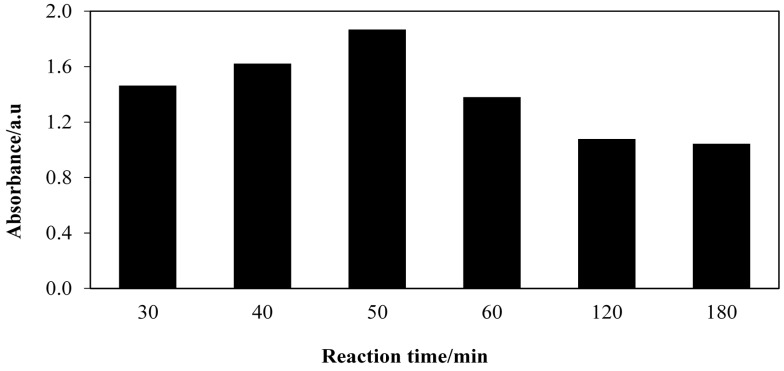
The effect of reaction time on the aminolysis reaction at 364 nm. Reaction conditions: Crude palm oil, 6 mmol; hydroxylamine hydrochloride, 1 mmol; CAL-A lipase, 20.0 mg; hexane, 3 mL; deionized H_2_O, 3 mL; temperature, 70 °C; shaking rate, 150 rpm.

Rapid response time is one of the crucial parameters in the proposed detection of free fatty acids as it determines the efficiency of the developed system. Hence, in this study various aminolysis reaction times were investigated in order to obtain the most rapid colour changes. Different reaction times ranging from 30 min up to 180 min were studied while other parameters were kept constant. Figure 2 shows the absorbance spectra for the aminolysis of FFAs to FHAs using *Candida antarctica* lipase A (CAL-A) at different reaction times. The detection wavelength was set at 364 nm. Figure 2 shows that the absorbance value increases up to 50 min of reaction. Further increases in the reaction time caused a decline in the absorbance. This phenomenon might be due to the fact excessive amounts of substrate are present at this rate, thus inhibiting the reaction. Hence, 50 min was chosen as the optimum reaction time since it gave the highest absorbance in the UV/Vis spectra.

### 2.3. Effect of Mole Ratio of Reactants

In enzymatic reactions the mole ratio of reactants is one of the most important parameters to produce high yields of product to react with V(V). Here, the amount of hydroxylamine hydrochloride was varied between 1 mmol and 3 mmol, while the amount of CPO was kept constant at 6 mmol. The colour intensity of the complex increases as the mole ratio of hydroxylamine hydrochloride increases. Theoretically, by increasing the mol ratio of reactants, the recovery of the product (FHA) available for reaction with V(V) increases. A similar trend was also reported in the literature [14,15,16]. In this study, 1 mmol of hydroxylamine hydrochloride to 2 mmol of CPO was selected as the optimum mole ratio of reactants since it gave the highest absorbance.

### 2.4. Effect of Temperature

**Figure 3:**
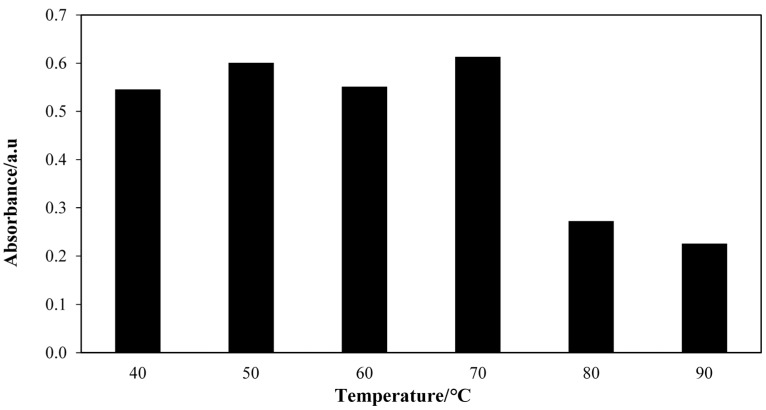
Absorbance maxima for aminolysis reactions at different reaction temperatures. Reaction conditions: Crude palm oil, 6 mmol; hydroxylamine hydrochloride, 3 mmol; CAL-A lipase, 20.0 mg; hexane, 3 mL; deionized H_2_O, 3 mL; shaking rate, 150 rpm; reaction time, 50 min.

As the enzymatic aminolysis reaction is an exothermic reaction, the temperature of the reaction is an important parameter to produce high throughput of product. Herein, the reaction temperature was investigated in the range of 40 °C to 90 °C while other parameters were kept constant. Rapid colour changes during complexation with 300 µL of V(V) occurred at 70 °C. Figure 3 shows a maximum absorptivity at the reaction temperature of 70 °C compared to others. This could be explained theoretically by the notion that at higher temperature, molecules move faster with higher energy and more successful collisions occur between them, and hence the rate of reaction increases. However, the absorptivity declined at temperatures higher than 70 °C due to the deactivation of the lipase which takes place at 80 °C. The result was in contrast with the research reported by Servat *et al*. [13] where the optimum reaction temperature was found to be in the range of 30–45 °C.

### 2.5. Effect of Enzyme

#### 2.5.1. Effect of Enzyme Mass

**Figure 4:**
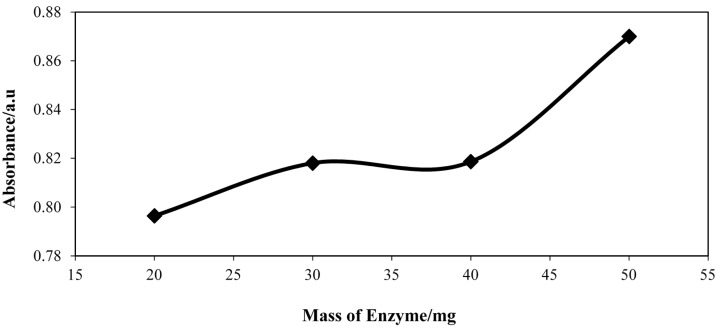
Absorbance spectra for FHA-V(V) complex after aminolysis of FFAs to FHAs using different amounts of enzyme. Crude palm oil, 6 mmol; hydroxylamine hydrochloride, 3 mmol; types of lipase; CAL-A, hexane, 3 mL; deionized H_2_O, 3 mL; V(V) ion solution, 300 µL; shaking rate, 150 rpm; reaction time, 50 min.

In this work, the amount of enzyme used for the synthesis of FHAs was optimized. Experiments were carried out by using different amounts of enzyme ranging from 20 mg to 50 mg during the aminolysis reaction. Previous research has reported that the yield of FHAs is increased when the amount of lipase enzyme is increased [13]. When a high concentration of enzymes is used, the time of reaction can be greatly reduced [15], thus rapid detection can be obtained. Figure 4 shows that, when the mass of enzyme is increased, the absorbance of the FHA-V(V) complex also increased.

A high absorbance value indicates high color intensity for the FHA-V(V) complex formed. When the intensity of FHA-V(V) complex is high, it represents a high amount of FFA in the sample. A similar trend has also been reported by Suhendra *et al*. [14] and Vaysse *et al*. [15].

#### 2.5.2. Effect of Enzyme Type

**Figure 5:**
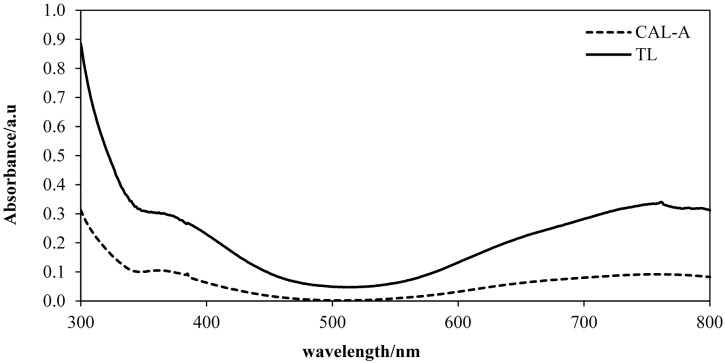
Absorbance spectra for FHA-V(V) complex after the aminolysis reaction using different types of enzymes. Reaction conditions: Crude palm oil, 6 mmol; hydroxylamine hydrochloride, 2 mmol; CAL-A lipase, 20.0 mg; TL lipase, 20.0 mg; hexane, 3 mL; deionized H_2_O, 3 mL; V(V) ion solution, 300 µL; temperature, 70 °C; shaking rate, 150 rpm; reaction time, 60 min.

**Figure 6:**
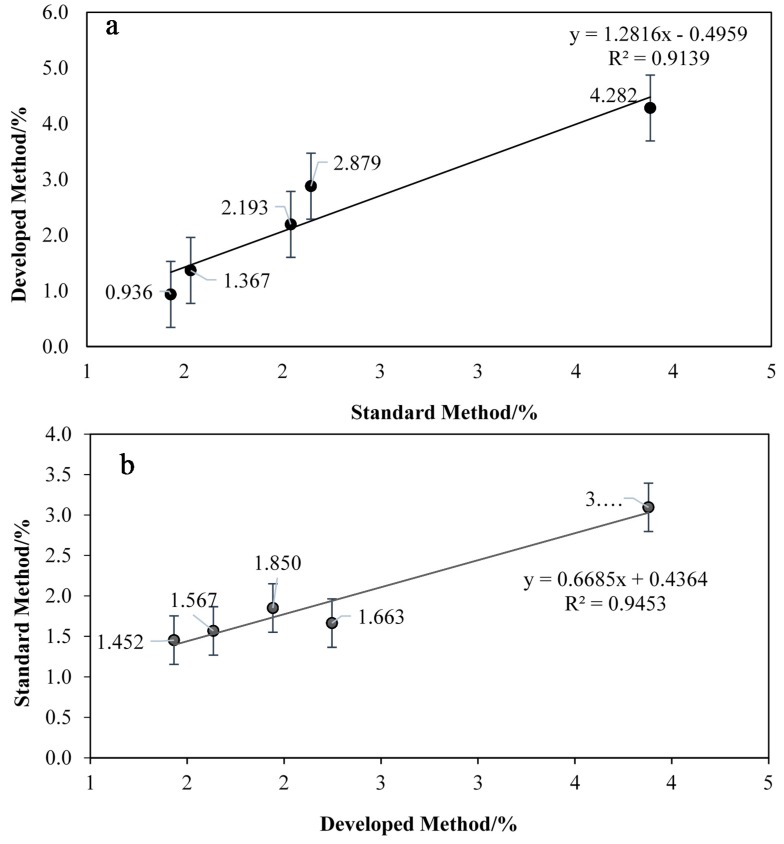
Correlation of FFA concentration between developed method and MPOB standard titration method using (**a**) *Candida antarctica* A (CAL-A) and (**b**) *Thermomyces lanuginose* (TL) lipases as catalyst during aminolysis of FFAs to FHAs.

In this study two types of enzymes were used, namely *Candida Antarctica* A lipase (CAL-A) and *Thermomyces lanuginose* lipase (TL). These enzymes are thermostable in nature and able to tolerate extreme reaction conditions [27,28]. Figure 5 shows that TL gave better absorbance for the FHA-V(V) complex compared to CAL-A. The colour changes were detected at 364 nm. High absorbance indicates more FHA was produced during the aminolysis reaction using TL, therefore more FHA was bound with V(V) to form the complex which exhibits a high color intensity, thus showing a high FFA content. The correlation study between the developed and standard methods was carried out by using both enzymes for further confirmation. Two sets of five unknown concentrations of FFA in CPO samples were tested using the developed method. The results obtained were compared with the MPOB standard method using manual titration. The correlation of the developed method using both enzymes is shown in Figure 6a,b.

Figure 6 shows the correlation between the developed method and the MPOB standard titration method for the detection of FFA in CPO using (a) *Candida antarctica* A (CAL-A) and (b) *Thermomyces lanuginose* (TL) lipases during aminolysis of FFAs to FHAs. A good correlation (R^2^ = 0.9453) was found for the MPOB standard titration method and the developed method using TL as catalyst. Different species of lipase give different temperature, pH stability, positional and stereoisomer specificity characteristics [29]. In this case, TL shows better thermostability characteristic compared to CAL-A, whereby it is quite stable and able to tolerate extreme reaction conditions, thus producing better results [30]. Therefore, TL was selected as the best enzyme for this reaction.

### 2.6. Effect of Different Concentrations of V(V)

**Figure 7:**
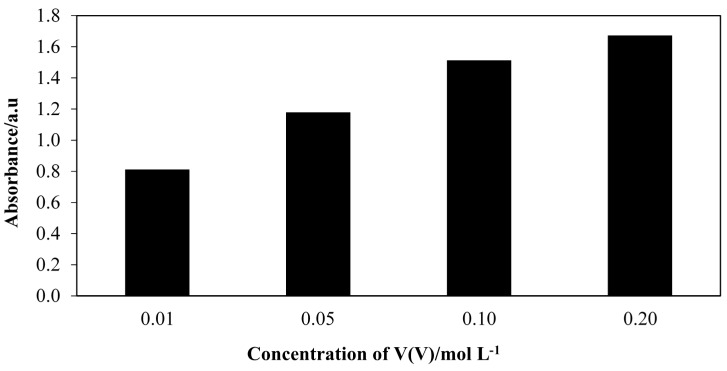
Absorbance spectra for FHA-V(V) complex after aminolysis reactions using different concentrations of V(V) ion solution. Reaction conditions: Crude palm oil, 6 mmol; hydroxylamine hydrochloride, 2 mmol; TL lipase, 20.0 mg; hexane, 3 mL; deionized H_2_O, 3 mL; temperature, 70 °C; shaking rate, 150 rpm; reaction time, 60 min.

Figure 7 shows the absorbance spectra for FHA-V(V) complex after the aminolysis reaction using different concentrations of V(V) ion solution. The results show a uniform trend whereby as the concentration of V(V) increases, more V(V) is bound to FHAs to produce complex, thus increasing the colour intensity of the absorbance.

## 3. Experimental Section

### 3.1. Materials and Reagents

Crude palm oil (CPO) was supplied by Sime Darby (Labu, Malaysia). Hydroxylamine hydrochloride (assay > 99%) and palmitic acid (C_16_H_32_O_2_), 98% were purchased from Acros Organics (Shah Alam, Malaysia). Two types of immobilized lipases, namely *Candida Antarctica* A (>1.5 U/mg) and *Thermomyces lanuginose* (>25 U/mg) were purchased from Sigma-Aldrich (Petaling Jaya, Malaysia) which appear in solid form. Vanadium (V) oxide (V_2_O_5_) was purchased from Aldrich (Petaling Jaya, Malaysia). Potassium hydroxide (KOH), hydrochloric acid (HCl) and 2-propanol were purchased from R & M Chemicals (Semenyih, Malaysia) and hexane was purchased from HmBG Malaysia (Kuala Lumpur, Malaysia). All chemicals were of analytical grade. A stock solution (0.1 mol·L^−1^) of V(V) was prepared by dissolving 1.8188 g of V_2_O_5_ in 100 mL of concentrated HCl.

### 3.2. Apparatus

A UV/VIS Spectrometer (Lambda 35, Perkin Elmer, Waltham, MA, USA) connected to a PC was used for absorbance measurements. The wavelength range was 190 nm to 900 nm for every sample analyzed.

### 3.3. General Procedure

FFAs were converted into FHAs based on the method reported by Suhendra *et al.* [14] with some modifications. The experiment was carried out by mixing the reactants, which included 6.0 mmol of crude palm oil dissolved in 3 mL of hexane, 2.0 mmol of hydroxylamine hydrochloride dissolved in 3 mL of deionized water, and 20 mg of immobilized lipase enzyme that were then shaken in a 100 mL flask sealed using Teflon film in water bath shaker at 150 rpm and 70 °C. In order to separate the product from the mixtures, the enzyme was filtered. The mixture consists of two layers, organic (top layer) and aqueous (bottom layer) phases that were easily separated. The organic phase containing FHA was pipetted out while the aqueous layer was discarded. The organic layer was further used for the complexation reaction. Afterwards, FHA derivatives in hexane were mixed with V(V) ion solution and shaken until the colour of the solution changed. The colour changes were observed to indicate the presence of FHAs in palm oil. The product formed from the reaction of FHA and metal solution is proportional to the FFA content [16]. Qualitative identification of FHA was carried out by naked eye observations of the intensity of the coloured solution due to the complex formation.

### 3.4. Correlation Study for the Developed Method with the MPOB Standard Titration Method

#### 3.4.1. Preparation of Crude Palm Oil (CPO) Stock Samples

Sample preparation method for correlation study was carried out following the method developed by Ali and Abdulrahman [31] with some modifications. CPO samples (25 g, molar mass = 858 g/mol) were dissolved in 2-propanol (50 mL). The sample was heated until the sample was homogenized and made up to the mark with 2-propanol. This sample was then used to prepare stock and standard palmitic acid solutions.

#### 3.4.2. Preparation of Stock Palmitic Acid Solution (100 a.d.)

Stock solutions of palmitic acid was prepared by dissolving palmitic acid (10.0 g) in CPO stock sample prepared above (100 mL). The solution was heated until it was homogenized and made up to the mark with CPO stock, and stored at room temperature. This solution was used to prepare working calibration curve samples by spiking a selected amount of palmitic acid stock into CPO stock sample. Table 1 shows the summary of the sample preparations for the working calibration curve sample.

**Table 1:** Sample preparation for the calibration curves.

Concentration Required, a.d.	Volume of Palmitic Acid Stock, mL	Volume of CPO Stock, mL	Total Volume Solution, mL
0.5	0.05	9.95	10.00
1.0	0.10	9.90	10.00
2.0	0.20	9.80	10.00
5.0	0.50	9.50	10.00
10.0	1.00	9.00	10.00
20.0	2.00	8.00	10.00
40.0	4.00	6.00	10.00
80.0	8.00	2.00	10.00
100.0	10.00	0.00	10.00

#### 3.4.3. MPOB Standard Titrimetric Method

The degree of acidity in CPO was determined using the MPOB standard titration method [8]. The free acids are expressed as a percentage of FFAs. Samples were dissolved in hot 2-propanol (50 mL) and 1.0% *w*/*v* phenolphthalein in 2-propanol (0.5 mL) was added to the sample, which was titrated against 0.1M potassium hydroxide (KOH) until the first pink colour appears and was persistent for 30 s.

#### 3.4.4. Preparation of Samples for the Correlation Study

Five unknown FFA concentration samples were prepared for the correlation study. The developed method for aminolysis of FFAs to FHAs and the MPOB standard titration method were carried out for the unknown samples. Percentages of FFAs obtained from both methods was correlated for validation of the data.

## 4. Conclusions

In this paper, an environmentally friendly enzymatic method for the detection of free fatty acids (FFAs) in crude palm oil was described where less chemical solvent is used during the whole operation and only 3 mL of solvent is needed for each sample tested. The proposed method is a modification of the one described in a previous report [14] where the product FHAs can be synthesized in a shorter time. It also offers simplicity in product separation where the FHAs could be pipetted out directly from the mixture. Moreover the immobilized enzyme (TL) is stable at high temperature during the reaction, and thus the reaction could be completed at a short time. Although the manual titration method can be completed in less than 50 min, it requires high amount of solvent (*ca*. 50 mL for each sample tested) during the operation. Based on the experimental data, the optimum conditions for the conversion of FFAs to FHAs is by shaking the mixture of reactants at 150 rpm in a water bath shaker at 70 °C in the presence of *Thermomyces lanuginose* lipase (TL) for 50 min of reaction and using a 2:1 mol ratio of crude palm oil:hydroxylamine hydrochloride. The developed method for detection of FFAs in CPO using immobilized *Thermomyces lanuginose* lipase (TL) as catalyst for the aminolysis process gives good agreement with the MPOB standard titration method (R^2^ = 0.9453) based on five data points.
